# Point Prevalence Surveys of Antimicrobial Prescribing in a Non-Acute Care Hospital in Saitama Prefecture, Japan

**DOI:** 10.1155/2022/2497869

**Published:** 2022-03-25

**Authors:** Noriomi Ishibashi, Ines Pauwels, Yuki Tomori, Yoshiaki Gu, Takefumi Yamaguchi, Takahiro Handa, Minoru Yamaoka, Daisuke Ito, Takehiko Sakimoto, Takuma Kimura, Kouichi Takizawa, Ryota Sato, Takahiro Sakashita, Akira Ooyama, Ann Versporten, Herman Goossens, Norihito Tarumoto, Shigefumi Maesaki, Norio Tanahashi

**Affiliations:** ^1^Department of Infectious Disease and Infection Control, Saitama Medical University, 38 Morohongo, Moroyama, Iruma-gun, Saitama 350-0495, Japan; ^2^Department of Internal Medicine, Maruki Memorial Medical and Social Welfare Center, 38 Morohongo, Moroyama, Iruma-gun, Saitama 350-0495, Japan; ^3^Laboratory of Medical Microbiology, Vaccine & Infectious Disease Institute, Faculty of Medicine and Health Science, University of Antwerp, Universiteitsplein, Universiteitsplein 1, 2610 Wilrijk, Belgium; ^4^Department of Gastroenterology and Hepatology, Saitama Medical University, 38 Morohongo, Moroyama, Iruma-gun, Saitama 350-0495, Japan; ^5^Department of Infectious Diseases, Graduate School of Medical and Dental Sciences, Tokyo Medical and Dental University, 1-5-45 Yushima, Bunkyo, Tokyo 113-8510, Japan; ^6^Department of Respiratory, Saitama Medical University, 38 Morohongo, Moroyama, Iruma-gun, Saitama 350-0495, Japan; ^7^Department of General Internal Medicine, Saitama Medical University, 38 Morohongo, Moroyama, Iruma-gun, Saitama 350-0495, Japan; ^8^Department of Endocrinology and Diabetes, Saitama Medical University, 38 Morohongo, Moroyama, Iruma-gun, Saitama 350-0495, Japan; ^9^Department of Digestive Tract and General Surgery, Saitama Medical Center, Saitama Medical University, 1981 Kamoda, Kawagoeshi, Saitama 350-8550, Japan; ^10^Department of Pharmacy, Maruki Memorial Medical and Social Welfare Center, 38 Morohongo, Moroyama, Iruma-gun, Saitama 350-0495, Japan; ^11^Department of Neurology, Saitama Medical University International Medical Center, 1397-1, Yamane, Hidaka, Saitama 350-1298, Japan

## Abstract

**Background:**

The global point prevalence survey (Global-PPS) is the standard for the surveillance of prescribed antimicrobials among inpatients and provides data for the development of hospital antimicrobial stewardship programs.

**Aim:**

To evaluate the prevalence and quality of antimicrobial prescriptions using the universally standardized Global-PPS protocol in a non-acute care hospital in Saitama Prefecture, Japan.

**Methods:**

Antimicrobial prescriptions for inpatients, staying at the hospital overnight, were surveyed on three separate week days in November 2018, January 2019, and May 2019. Information on the prescribed antimicrobials on the survey target day was obtained from the in-hospital pharmacy. Survey data were collected by physicians, based on the extracted information. Patient information was anonymized and entered in the Global-PPS Web application by physicians. We report the antimicrobial use prevalence, the indication for prescription, diagnosis, the most prescribed antimicrobials, and a set of quality indicators related to antimicrobial prescribing.

**Results:**

In total, 6.7% of the surveyed inpatients (120/1796) were prescribed antimicrobials on the survey day. Sulfamethoxazole/trimethoprim was the most commonly prescribed, with 20.0% of systemic antibiotic prescriptions (ATC J01). Of all antibiotics for systemic use, up to 58.4% were Watch antibiotics, as defined by the World Health Organization AWaRe classification. The most prescribed group of systemic antibiotics was non-penicillin beta-lactam antibiotics (34.4%), followed by penicillin antibiotics in combination with beta-lactamase inhibitors (25.6%), and sulfonamides with trimethoprim (20.8%). Healthcare-associated infections and medical prophylaxis were the most common indications reported in 69.3% and 26.3% of prescriptions, respectively. The most common diagnosis for systemic antibiotic prescriptions was pneumonia (49.6%). Reasons for antimicrobial prescriptions were indicated in the medical records for 67.1% of prescriptions, and the stop/review date was documented to be 50.3%. Compliance with local guidelines reached 66.7%.

**Conclusions:**

This study highlights important challenges related to antimicrobial prescription in a highly specific, non-acute care patient population.

## 1. Introduction

Antimicrobial resistance is a global health concern [1] and requires coordinated action at the local, national, and global levels [2]. Uniform and standardized surveillance of antimicrobial use is critical for effective antimicrobial stewardship (AS) not only in tertiary referral hospitals but also in smaller secondary, primary, and specialized hospitals [3]. The first global point prevalence survey of antimicrobial consumption and resistance (Global-PPS) was conducted at 303 hospitals across 53 countries in 2015 and primarily involved tertiary hospitals (including acute care facilities or university hospitals) [4]. The WHO AWaRe classification categorizes antibiotics used worldwide into 4 groups (Access, Watch, Reserve, and Not Recommended) according to their role in empiric treatment of common infections and the risk of selecting for antibiotic resistance [5]. Results from a study using worldwide Global-PPS data, collected between 2015 and 2018, show an overall high use of antibiotics from the Watch category (i.e., the antibiotics with a higher risk of resistance selection), with substantial differences at country level [6]. Similarly, results from specific Global-PPS studies in the Asian region vary, depending on the hospital-specific antimicrobial prescribing practices, and other country or hospital characteristics [7, 8]. Surveys using Global-PPS have mainly focused on tertiary medical institutions, and no study has been reported for more specialized, non-acute care settings. In addition, the cumulative antibiogram for inpatients from the Maruki Memorial Welfare and Medical Center showed that the sensitivity to ciprofloxacin in 2018 was 46.7% for *Escherichia coli* and 69.7% for *Klebsiella pneumoniae*. Given these high isolation rates of antimicrobial-resistant bacteria, it was decided to conduct a hospital-wide point prevalence survey. This study aimed to evaluate the prevalence and quality of antimicrobial prescriptions for inpatients. The results will help identify targets for improvement of antimicrobial prescribing and inform the development of antimicrobial stewardship activities, an approach that has proven successful in other countries and settings [8–10].

## 2. Methods

### 2.1. Study Design and Setting

A hospital-wide point prevalence survey on antimicrobial use, following the Global-PPS methodology and tools, was conducted on a weekday in November 2018, January 2019, and May 2019 at the Maruki Memorial Welfare Medical Center. The study hospital is located in the western part of Saitama Prefecture, Japan, and has a highly specific patient population. The inpatient wards are divided into psychiatric wards (PWs) and general wards (GWs). Most of the GW inpatients are older individuals with dysphagia or are hospitalized for rehabilitation due to cerebrovascular disorders. Furthermore, some patients required long-term fluid management due to cardiac failure or pulmonary disease. Patients requiring indwelling intravascular catheters and urinary catheters also required long-term fluid management. No antimicrobial stewardship interventions were implemented during the entire duration of the study.

### 2.2. Data Collection and Analysis

One weekday was selected for each of the three survey periods (November 2018, January 2019, and May 2019, further abbreviated as first, second, and third period, respectively). All inpatients hospitalized at 8 am on the day of the survey were included. Patient-level antimicrobial prescription information was collected for all inpatients on antimicrobials at 8 am on the day of the survey. Outpatients and day admissions were excluded. Further details on the Global-PPS protocol have been described elsewhere [4]. The medical data of patients prescribed antimicrobials were obtained from the hospital pharmacy in spreadsheet format, verified against the medical records, and extracted based on the Global-PPS surveillance sheet by physicians (supplementary file 1). The physicians performing data collection were trained in advance on the Global-PPS methodology, and the data were checked for uniformity by the principal investigator. The following patient-level data were collected: patient demographics, biomarkers, culture collection status, the prescribed antimicrobial, dose and frequency, route of administration, diagnosis, and type of indication. Prescription quality indicators included reason indicated in notes, compliance with local guidelines, and stop/review date documentation. We merged all 3 data points and described the overall results. In addition, we report the results for the 3 points separately. The antimicrobial use prevalence was estimated as the ratio of the number of patients prescribed antimicrobials to the total number of inpatients in each ward surveyed. Antimicrobials were classified using Anatomical Therapeutic Chemical Classification (ATCC) code levels. Systemic antimicrobials (ATCC J01) were further categorized using the AWaRe classification of the World Health Organization [5]. We subsequently calculated Access, Watch, and Reserve percentages, as well as the Access-to-Watch ratio. The indications for prescriptions were classified as community-acquired infections (CAI), hospital-acquired infections (HAI), medical prophylaxis (MP), surgical prophylaxis (SP), other (OTH), and unknown (UNK). The most prescribed indications were reported, and for MP, the underlying disease was noted for each period. A Web-based application designed by the University of Antwerp (https://www.global-pps.com) was used for data entry, validation, and reporting.

### 2.3. Ethical Approval

This study was approved by the institutional review boards and ethics committees of the Maruki Memorial Welfare and Medical Center (No. 24) and conformed to the tenets of the Declaration of Helsinki (The Code of Ethics of the World Medical Association).

## 3. Results

### 3.1. Patient and Hospital Demographics

On survey days, 1796 beds out of 1872 were occupied; the bed occupancy rate was 95.9% on average (rates for PWs and GWs were 96.7% and 94.0%, respectively; Table 1). The average age of all inpatients was 72.6 y (range for GW inpatients: 38–93 y; PW inpatients: 40–95 y).

### 3.2. Antimicrobial Use Prevalence

Over the three survey periods, a total of 137 antimicrobials were prescribed to inpatients. The total hospital prevalence of antimicrobial use was 6.7% (120 of 1796 inpatients); within this, the prevalence for the PWs was 4.4%, and that for the GWs was 12.5%. Within the PWs, the prevalence of antimicrobial use was 20.5% in the psychiatric-medical combined ward and ranged from 0% to 5.9% in the other PWs. Looking at the different survey periods, the hospital-wide antimicrobial prevalence was 4.8% in the first period (PW: 3.7%; GW: 7.7%), 8.8% in the second period (PW: 5.7%; GW: 17.2%), and 6.5% in the third period (PW: 3.9%; GW 12.9%).

### 3.3. Most Commonly Prescribed Antimicrobials

**Figure 1:**
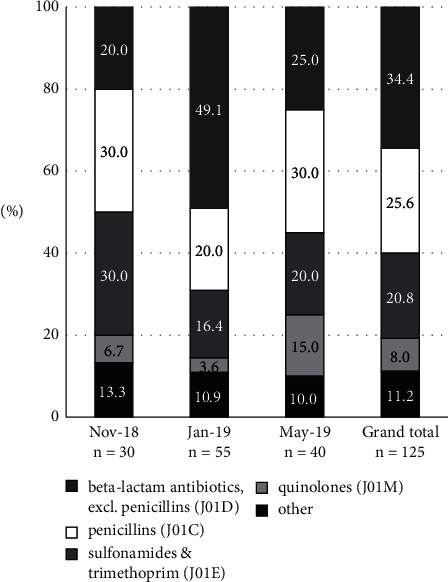
Most prescribed antibiotic (J01) groups according to the WHO/ATCC (level 3) at the Maruki Memorial Welfare and Medical Center, Japan.

**Figure 2:**
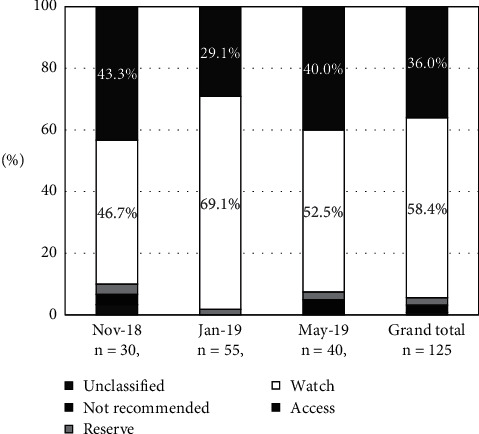
AWaRe classification for systemic use antibiotics (J01) during three survey periods in November 2018, January 2019, and May 2019 at the Maruki Memorial Welfare and Medical Center, Japan.

Non-penicillin beta-lactam antibiotics (J01D) were the most commonly prescribed group of systemic antibiotics (34.4% of total prescribed; 43 out of 125), the majority of which were third-generation cephalosporins (23.2% of total prescribed; 29 out of 125) (Figure 1). Penicillins (J01C) were also commonly prescribed (25.6%; 32 out of 125); 22.4% (28 out of 125) of all systemic antibiotics comprised combinations of penicillin with a beta-lactamase inhibitor. From November 2018 to January 2019, the proportion of prescriptions for penicillin antibiotics (J01C) dropped from 30% to 20%. Concomitantly, the prescription of non-penicillin beta-lactam antibiotics (J01D) increased from 20% to 49.1%. Combinations of sulfonamides with trimethoprim (J01E) accounted for 20.8% (26 out of 125) of all antibiotic prescriptions in January 2019. The most commonly prescribed systemic antibiotics were sulfamethoxazole/trimethoprim (20.0%; 25 of 125), sulbactam/ampicillin (12.0%; 15 of 125), and tazobactam/piperacillin (9.6%; 12 of 125) (Table 2). The total hospital-wide AWaRe percentages for systemic antibiotics (J01) were composed of 36.0% Access, 58.4% Watch, 2.4% Reserve, and 2.4% were “Not Recommended” (Figure 2). In the second survey period, Access prescriptions were the lowest (29.1%), and Watch prescriptions were the highest (69.1%). The overall Access-to-Watch ratio was 0.62.

### 3.4. Reasons for Antimicrobial Prescribing

In this study, 71.5% of antimicrobials (98 out of 137) were prescribed for treatment and 27.0% (37 out of 137) were for prophylaxis (Table 3). The most common diagnosis among patients prescribed antimicrobials was pneumonia (49.6%, 62 out of 125). Sulfamethoxazole/trimethoprim was mainly used for medical prophylaxis, accounting for 69.4% of all medical prophylactic prescriptions (25 out of 36). Numerous patients who were prescribed sulfamethoxazole/trimethoprim had a history of autoimmune disease, with 87.5% of them receiving systemic steroids (21 out of 24). Underlying diseases included interstitial pneumonia (23.1%), systemic lupus erythematosus (19.4%), and ulcerative colitis (14.8%).

### 3.5. Quality Indicators of Antimicrobial Use

**Figure 3:**
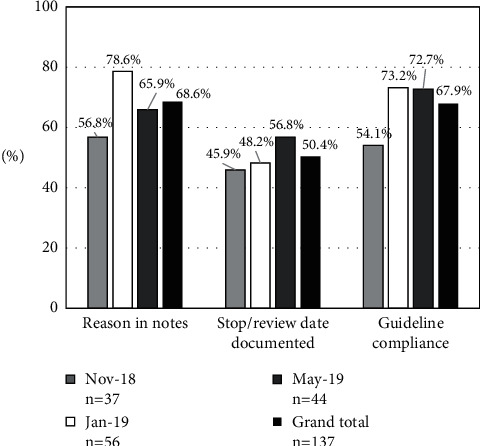
Quality indicators for antimicrobial prescriptions during three survey periods in November 2018, January 2019, and May 2019 at the Maruki Memorial Welfare and Medical Center, Japan.

Overall, the reason for prescription was documented in the notes for 68.6% of the antimicrobial prescriptions. Stop/review date was documented for 50.4% of prescriptions, and guideline compliance was 67.9% for all antimicrobial prescriptions. Quality indicators for each of the survey periods separately are shown in Figure 3.

## 4. Discussion

The overall antimicrobial use prevalence observed in the study was 6.7%, which was substantially lower than the 2015 global data (34.4%) and the regional data for East and South Asia (37.5%) [4]. The reason for this is the difference in hospital background: 51.7% and 36.6% of the hospitals in the 2015 Global-PPS were tertiary and secondary acute care hospitals, respectively. However, we report the results of a specialized hospital, with a highly specific patient population. Up to 72.0% of the patients included in our study were psychiatric patients (1294 admitted on psychiatric wards out of 1796 included patients), which clearly influenced the antimicrobial prescription patterns. A survey conducted by the Japanese National Center for Global Health and Medicine found that antimicrobial use prevalence in long-term care facilities in Japan was 1.5% across multiple medical care facilities [11]. Another study reported an antimicrobial use prevalence of 6.3% in long-term care facilities in England [12], which is similar to our findings.

The percentage of prescriptions for sulfamethoxazole/trimethoprim at the Maruki Memorial Welfare Medical Center was high, representing 69.4% of all antimicrobial prescriptions for medical prophylaxis. This is because the center uses steroids and immunosuppressive drugs to treat patients acutely for collagen disease and ulcerative colitis, as a university hospital. Prednisolone is then used chronically for maintenance. This was also the case for patients who underwent joint surgery for rheumatoid arthritis and were transferred to a hospital for postoperative rehabilitation. Sulfamethoxazole-trimethoprim is typically prescribed regularly to prevent *Pneumocystis* pneumonia in non-HIV immune-compromised hosts. However, administration may increase the risk of pancytopenia and hypokalemia [13].

Penicillin antibiotics (J01C) were also commonly used, mainly in combination with *β*-lactamase inhibitors. This can be explained by the unstable supply chain of antimicrobials during this period. Shortages of sulbactam/ampicillin in Japan started in June 2018 and continued for over a year because of the inability to secure active pharmaceutical ingredients that met quality standards. Sulbactam/ampicillin is commonly used to treat pneumonia at the Maruki Memorial Welfare Medical Center. Broad-spectrum antibiotic use for pneumonia is high worldwide, and clavulanate/amoxicillin is frequently prescribed [6]. However, the drip injection form of clavulanate/amoxicillin has not been adopted in Japan; only oral formulations are available. This may explain why the Maruki Memorial Welfare Medical Center had fewer clavulanate/amoxicillin prescriptions than sulbactam/ampicillin and tazobactam/piperacillin prescriptions in general. This may also explain the need to resort to cephem antimicrobials as an alternative.

Next, the antimicrobial agents were analyzed according to the WHO AWaRe classification. The WHO recommends that Access antibiotics account for at least 60% of the national antimicrobial consumption. This target has been developed to support antimicrobial use monitoring and to improve access to essential medicines, ultimately reducing the emergence and spread of antimicrobial resistance [14]. In the Maruki Memorial Welfare Medical Center, Access prescriptions accounted for 36.0% of the antibiotics for systemic administration, and 58.4% of the antibiotics corresponded to the more wide-spectrum Watch antibiotics. A PPS analysis of 69 countries [6] estimated ∼35% Access antibiotic prescriptions in East and South Asian hospitals; Watch antibiotics accounted for ∼60%, which was similar to our findings. The observed high use of Watch antibiotics may be because they are typically prescribed for hospital-acquired pneumonia, given the need to empirically cover *Pseudomonas* and extended spectrum beta-lactamase (ESBL)-producing bacteria. Additionally, since surgical procedures are not performed in this hospital, antibiotics are not administered for surgical prophylaxis, which also contributes to the low observed proportion of Access prescriptions (compared with acute care hospitals).

To check the prescription balance between Access and Watch in the AWaRe classification, the Access-to-Watch ratio was evaluated, and the figure in our hospital was 0.62. This value was lower than the value obtained in many countries in the worldwide analysis, which averaged 0.7. Country-level Access-to-Watch ratios in this global study ranged from 0.1 to 2.1 [6].

Indications for antimicrobial prescription were mainly classified as HAI (95 : 69.3%) and MP (36 : 26.2%). Antimicrobial prescriptions for CAI (3 : 2.2%), SP (1 : 0.7%), and OTH (1 : 0.7%) were low. In 2015, CAI accounted for 45.6% of prescriptions at the global level and 36.9% in East and South Asia [4], while we estimated only 2.2%. Again, the reason for this is likely that our study mainly included patients in long-term psychiatric care and non-acute care patients in internal medicine, and there were no emergency patients included.

The most common diagnosis was pneumonia, which accounted for 49.6% (62 out of 125) of antibiotic prescriptions. In our hospital, broad-spectrum combinations of penicillin and a beta-lactamase inhibitor were first prescribed as empirical therapies for hospital pneumonia. At that time, it was a practice in antimicrobial drug treatment to perform a sputum culture, confirm the culture result, and refer to the culture result prior to prescribing a drug; however, there was no microbiology test department in the hospital, and a written report was sent back to the hospital from an external laboratory. Therefore, it took time to receive feedback for the test result and there were many situations in which the report of the bacterial test could not contribute to the de-escalation of the antimicrobial agent. Since February 2020, bacterial test reports have been fed back in the form of electronic medical reports; thus, it is possible to estimate bacterial species and make changes in antimicrobial agents early during the course of treatment. Future PPS measurements will be conducted to verify how antimicrobial prescriptions have changed since these online reports and electronic medical services have been made available.

Quality indicators for antimicrobial prescribing included reasons for prescriptions in notes, guideline compliance, and stop/review documentation. In general, the medical records were detailed with regard to medical treatment steps, selection of antimicrobial agents, and administration start criteria, but there were few records with a stop criterion or review, and the status of termination was unclear. Documentation of a stop/review date was low, at only 50.4% of all prescriptions. In the 2015 global analysis, 38.3% of the antimicrobial prescriptions globally and 19.8% of prescriptions in East and South Asia had a recorded date for stopping or reviewing the antimicrobial regimen [4]. According to the quality indicators measured in other countries and regions, suboptimal documentation of the stop/review date (<70%) is a problem faced by multiple hospitals of all sizes [4, 7, 8]. A possible explanation for why the documentation of antimicrobial therapy in patient notes was suboptimal would be that many part-time doctors were engaged in medical care at the time of the survey.

### 4.1. Limitations

This survey was conducted on three separate days at three distinct time periods; hence, the specific survey timing and patient population may have affected the results. Moreover, there is variation in the time between each of the surveys; two months and four months, respectively. Additionally, this hospital is a regional subacute care hospital; therefore, it does not provide the advanced medical care found in regional acute care hospitals and university hospitals (such as those that participated in the Global-PPS in 2015). Furthermore, 72% of the inpatients had psychiatric conditions; they could not be compared with other adult inpatients at tertiary care hospitals (who were assessed in previous surveys in Japan). In addition, antimicrobial prescriptions were grouped across the three periods. As our institution is a non-acute care hospital, often admitting patients for longer periods of time, some patients may have been included in more than one survey. Extracting information from medical records with missing data may have resulted in unclear prescription patterns. Furthermore, antimicrobial selection was only evaluated in accordance with the Global-PPS protocol, and the appropriate doses (based on body weight and renal function) were not evaluated.

## 5. Conclusion

This study shows the prevalence and quality of antimicrobial use for three periods at a specialized hospital in Saitama Prefecture, Japan. Likely due to the specific patient population, the prevalence of antimicrobial use and the proportion of antimicrobials prescribed for community-acquired infections were lower compared with the results of acute care hospitals in Asia. In the study hospital, antimicrobials were mainly used for hospital-acquired infections and medical prophylaxis. Future antimicrobial stewardship efforts will be directed at reducing the proportion of broad-spectrum Watch antibiotics used, improving the documentation of stop/review date in the medical charts, optimizing the system of audit and feedback by pharmacists and integrating microbiological results into the clinical decision-making process. We aim to continue to conduct repeated PPS measurements to track changes in antimicrobial prescribing and hope that the results of these consecutive surveys will lead to sustained behavioral changes in physicians' prescribing practices. As a future research outlook, we would like to increase the number of hospitals that can cooperate with the Global-PPS survey at specialized institutions in similar areas. In addition, we would like to implement and integrate results on antimicrobial drug prescriptions and quality indicators, obtained in the western part of Saitama Prefecture at multiple medical institutions, and utilize them as regional antimicrobial drug surveillance data.

## Figures and Tables

**Table 1:** Bed occupancy and antimicrobial use prevalence at the Maruki Memorial Welfare and Medical Center, Japan, in November 2018, January 2019, and May 2019 (GW = general ward; PW = psychiatric ward).

	18-Nov		19-Jan		19-May		Grand total	
Bed occupancy rate on total	97.1%		97.0%		93.8%		95.9%	
	(606/624)		(605/624)		(585/624)		(1796/1872)	
Average age	71		72.3		74.4		72.6	
Bed occupancy rate in department	94.9%	98%	91.6%	99.1%	95.5%	93%	94%	96.7%
	(169/178)	(437/446)	(163/178)	(442/446)	(170/178)	(415/446)	(502/534)	(1294/1338)
Prevalence of antimicrobial use	4.8%		8.8%		6.5%		6.7%	
	(29/606)		(53/605)		(38/585)		(120/1796)	
Prevalence of antimicrobial use by department type	7.7%	3.7%	17.2%	5.7%	12.9%	3.9%	12.5%	4.4%
	(13/169)	(16/437)	(28/163)	(25/442)	(22/170)	(16/415)	(63/502)	(57/1294)
	GW	PW	GW	PW	GW	PW	GW	PW

**Table 2:** Top five most commonly used antibiotics for treating inpatients at the Maruki Memorial Welfare and Medical Center, Japan, in November 2018, January 2019, and May 2019.

Top 5 most commonly used antibiotics for therapy (*n* = 95)^*∗*^		
Antibiotic	%	AWaRe class
Ampicillin and enzyme inhibitor	15.8	Access
Piperacillin and enzyme inhibitor	12.6	Watch
Cefcapene	11.6	Watch
Levofloxacin	8.4	Watch
Meropenem	6.3	Watch

^*∗*^Systemic antibiotic (J01) prescriptions for community-acquired and healthcare-associated infections (excludes prophylaxis).

**Table 3:** Indications for antimicrobial prescribing (percentage of antimicrobial prescriptions) at the Maruki Memorial Welfare and Medical Center, Japan, in November 2018, January 2019, and May 2019.

	18-Nov (*n* = 37) (%)	19-Jan (*n* = 56) (%)	19-May (*n* = 44) (%)	Grand total (*n* = 137) (%)
Community-acquired infections (CAI)	2.7	0.0	4.5	2.2
Healthcare-associated infections (HAI)	54.1	80.4	68.2	69.3
Medical prophylaxis (MP)	40.5	17.9	25.0	26.3
Surgical prophylaxis (SP)	0.0	0.0	2.3	0.7
Other indications	2.7	0.0	0.0	0.7
Unknown indications	0.0	1.8	0.0	0.7

## Data Availability

The data used to support the findings of this study have not been made available because of ethics committee permission.

## Supplementary Materials

Supplementary Materials Supplementary file 1: Global-PPS data collection forms, version 2018.
