# Supplementary material for: Point Prevalence Surveys of Antimicrobial Prescribing in a Non-Acute Care Hospital in Saitama Prefecture, Japan
Source: Can J Infect Dis Med Microbiol. 2022 Mar 25;2022:2497869. doi: 10.1155/2022/2497869 (PMC8975653; doi:10.1155/2022/2497869)
Supplement: Supplementary Materials — Supplementary file 1: Global-PPS data collection forms, version 2018. [file 2497869.f1.pdf]

Global Point Prevalence Survey (PPS) – Year 2018 (GLOBAL-PPS)

Note: The aim of this GLOBAL-PPS is to find out what the physicians intend treating and not to base the diagnosis on any case definitions. To obtain this information the investigator should be looking at all [medical, nursing and drug prescription chart] patient records. If the information available is not sufficient surveyor/s may request additional information from nurses, pharmacists or doctors caring for the patient. Searching for information from other sources such a laboratory computer systems, phoning laboratories *etc.,* is not required. **At no point shall there be any discussion about the appropriateness (or lack thereof) of the prescribed medication. The ward staff MUST NOT feel evaluated at the individual level**.

Include in the survey: All patients who are receiving anti-infective agents (ATC codes: J01, J02, A07AA, P01AB, D01BA, J04A, J05 and P01B) and who are in the hospital at 8:00 am on the day of survey should be included in the study.

Prophylaxis: Include any patient who received one or more doses of anti-infective agents intended as surgical prophylaxis in the 24 h prior to 8:00 am on the day of the survey. Checking for any doses administered on the previous day/s will allow the surveyor to code the surgical prophylaxis as either 1 dose, 1 day (multiple doses within 24 hours) or >24hours.

Diagnosis Group: This information is obtained from Appendix II. The conditions are grouped by anatomical site and whether the indication (treatment intent) is prophylaxis or therapeutic.

Global Point Prevalence Survey (2018 GLOBAL-PPS)

Ward Form

Please fill in one form for each ward included in the PPS

**Date of survey**<br>(dd/mm/year)	____________/_________/__________________		
**Person completing form** (Auditor code)			
**Hospital name**			
**Ward Name**			
**Department Type:**<br>Place a tick against the type of department	**Paediatric departments:**<br>☐ **PMW** (Paediatric Medical Ward)<br>☐ **HO-PMW** (Haematology-Oncology PMW)<br>☐ **T-PMW** (Transplant (BMT/Solid) PMW)<br>☐ **PSW** (Paediatric Surgical Ward)<br>☐ **PICU** (Paediatric Intensive Care Unit)<br>**Neonatal departments:**<br>☐ **NMW** (Neonatal Medical Ward)<br>☐ **NICU** (Neonatal Intensive Care Unit)		**Adult departments:**<br>☐ **AMW** (Adult Medical Ward)<br>☐ **HO-AMW** (Haematology-Oncology AMW)<br>☐ **T-AMW** (Transplant (BMT/solid) AMW)<br>☐ **P-AMW** (Pneumology AMW)<br>☐ **ASW** (Adult Surgical Ward)<br>☐ **AICU** ([Adult] Intensive Care Unit)
**Mixed Department**	☐ **Yes** ☐ **No**		
**Activity:** Tick as appropriate.<br>➢ In case of mixed departments, tick all the encountered activities/specialities	☐ **Medicine**	☐ **Surgery**	☐ **Intensive Care**
**Total number of admitted patients** on the ward present at 8.00 am on day of PPS split up by activity.<br>➢ For mixed departments, fill the total number of patients corresponding to each of the encountered activities.			
**Total number of beds** on the ward present at 8:00 am on day of PPS split up by activity.<br>➢ For mixed departments fill in the total number of beds corresponding to each of the encountered activities.			

Include only inpatients "admitted before 08:00 hours" on the day of the PPS !

GLOBAL-PPS PATIENT Form (Please fill in one form per patient on antimicrobial treatment/prophylaxis)

Ward Name/code	Activity [1] (M, S, IC)	Patient Identifier [2]	Survey Number [3]	Patient Age [4]			Weight In kg, 2 decimals	Gender M or F
				Years (if ≥ 2 years)	Months (1-23 month)	Days (if <1 month)		

Antimicrobial Name [5]		1.		2.		3.		4.		5.	
**Single Unit Dose** [6]	**Unit** (g, mg, or IU) [7]										
**Doses/ day** [8]	**Route** (P, O, R, I) [9]										
**Diagnosis** [10] (see appendix II)											
**Type of indication** [11] (see appendix III)											
**Reason in Notes** (Yes or No) [12]											
**Guideline Compliance** (Y, N, NA, NI) [13]											
**Is a stop/review date documented?**(Yes/No)											
**Treatment** (E: Empirical; T: Targeted)											
The next section is to be filled in only if the treatment choice is based on microbiology data (Treatment=targeted) AND the organism is one of the following											
**MRSA** (Yes or No) [14]											
**MRCoNS** (Yes or No) [15]											
**VRE** (Yes or No) [16]											
**ESBL-producing Enterobacteriaceae** (Yes or No) [17]											
**3rd generation cephalosporin resistant Enterobacteriaceae non-ESBL producing or ESBL status unknown** (Yes or No)											
**Carbapenem-resistant Enterobacteriaceae** (Yes or No) [18]											
**ESBL-producing non fermenter Gram-negative bacilli** (Yes or No) [19]											
**Carbapenem-resistant non fermenter Gram-negative bacilli** (Yes or No) [20]											
**Targeted treatment against other MDR organisms** (Yes or No) [21]											

**Treatment based on biomarker data** (Yes or No)	0 Yes – 0 No				
**If yes, which biomarker** (CRP, PCT or other)[22]		**Type of biological fluid sample** (Blood/urine/other)		**Most relevant value of biomarker on the day of the PPS** Value	Unit (in µg/L, mg/L, …)[23]

[1] Activity: M=medicine (including Psychiatric cases, *etc*.), S=surgery (including orthopaedics, obstetrics and gynaecology, *etc*.), IC=intensive care

[2] Patient Identifier: A unique patient identifier that allows linkage to patient records at local level for more detailed audit. This unique identifier will not be included in the online database.

[3] Survey Number: A unique non-identifiable number given by WebPPS for each patient entered in the database. Leave blank but note down the number after the patient data has been recorded in the online database. The number is displayed once (and only) after the patient data has been recorded in the online database.

[4] Patient Age: If the patient is 2 years old or older, specify only the number of years, if between 1 and 23 months specify only the number of months, if less than 1 month specify the number of days.

[5] Antimicrobial Name: Insert generic name.

[6] Single Unit Dose: Numeric value for dose per administration (in grams, milligrams or IU).

[7] Unit: The unit for the dose (g, mg or IU)

[8] Doses/day: If necessary provide fractions of doses: (e.g., every 16h = 1.5 doses per day, every 36h = 0.67 doses per day, every 48h = 0.5 doses per day)

[9] Route: Routes of administration are: Parenteral (P), Oral (O), Rectal (R), Inhalation (I).

[10] See diagnoses groups list (Appendix II)

[11] See Indication codes (Appendix III)

[12] Reason in Notes: A diagnosis / indication for treatment is recorded in the patient's documentation (treatment chart, notes, etc.) at the start of antibiotic treatment (Yes or No)

[13] Guideline Compliance: Refers to antibiotic choice (not route, dose, duration etc) in compliance with local guidelines (Y: Yes; N: No; NA: Not assessable because no local guidelines for the specific indication; NI: no information because indication is unknown)

[14] Methicillin-resistant *Staphylococcus aureus* (MRSA)

[15] Methicillin-resistant coagulase negative staphylococci (MRCoNS)

[16] Vancomycin-resistant enterococci (VRE)

[17] Bacteria, producing extended-spectrum beta-lactamases (ESBL)

[18] Carbapenem-resistant *Enterobacteriaceae* (CRE) – enteric bacteria resistant to imipenem, meropenem or other carbapenems

[19] Nonfermenters: *Pseudomonas aeruginosa, Acinetobacter baumannii, Burkholderia spp., Stenotrophomonas maltophilia*

[20] Carbapenem-resistant Nonfermenters (CR-NF) – nonfermenters resistant to imipenem, meropenem or other carbapenems

[21] Multi-drug resistant (MDR) pathogens, others than the listed above.

[22] If treatment based on biomarker, specify which one: CRP (C-reactive protein), PCT (Procalcitonin) or Other (=lab-based culture and sensitivity result from a relevant biological sample)

[23] The unit for the biomarker CRP or PCT value expressed in mg/l, µg/L, ng/L, mg/dL, ng/dL, ng/mL, µg/mL, nmol/L. For a conversion calculator see: http://unitslab.com/node/67 (CRP) and http://unitslab.com/node/103 (procalcitonin).

Appendix I: Combination anti-infective agents

Combinations of an antibiotic and an enzyme inhibitor:
Ampicillin and enzyme inhibitor: report only ampicillin dose (J01CR01)
Amoxicillin and enzyme inhibitor: report only amoxicillin dose (J01CR02)
Ticarcillin and enzyme inhibitor: report only ticarcillin dose (J01CR03)
Piperacillin and enzyme inhibitor: report only piperacillin dose (J01CR05)
Imipenem and enzyme inhibitor: report only imipenem dose (J01DH51)
Panipenem and betamipron: report only panipenem (J01DH55)

Example:
Augmentin® 1.2g IV → 1g (amoxicillin) + 200mg (clavulanic acid), **report only 1 g**
Piperacillin® 4.5g IV → 4g (piperacillin) + 500mg (tazobactam), **report only 4 g**

Other combinations of multiple antimicrobial substances:
J01EE01 Sulfamethoxazole and Trimethoprim: **report the total amount of sulfamethoxazole and trimethoprim**
Example:
Co-trimoxazole 960mg: (sulfamethoxazole. 800mg + trimethoprim 160mg), **report 960mg**

Further information on agents included for the Global-PPS is available in the antimicrobial list. Only antimicrobial substance name need to be written down, NOT the ATC codes! (excel file - available at website under documents: Global-PPS_antimicrobial_list.xlsx)
http://www.global-pps.com/

Appendix II - Diagnostic codes (what the clinician aims at treating)

Site	Codes	Examples
**CNS**	**Proph CNS**	Prophylaxis for CNS (neurosurgery, meningococcal)
	**CNS**	Infections of the **C**entral **N**ervous **S**ystem
**EYE**	**Proph EYE**	Prophylaxis for Eye operations
	**EYE**	Therapy for Eye infections e.g., Endophthalmitis
**ENT**	**Proph ENT**	Prophylaxis for **E**ar, **N**ose, **T**hroat (**Surgical or Medical prophylaxis=SP/MP**)
	**ENT**	Therapy for **E**ar, **N**ose, **T**hroat infections including mouth, sinuses, larynx
**RESP**	**Proph RESP**	Pulmonary surgery, prophylaxis for **Resp**iratory pathogens e.g. for aspergillosis
	**LUNG**	Lung abscess including aspergilloma
	**URTI**	**U**pper **R**espiratory **T**ract viral **I**nfections including influenza but not ENT
	**Bron**	Acute **Bron**chitis or exacerbations of chronic bronchitis
	**Pneu**	**Pneu**monia or LRTI (lower respiratory tract infections)
	**TB**	Pulmonary TB (Tuberculosis)
**CVS**	**Proph CVS**	**C**ardiac or **V**ascular Surgery, endocarditis prophylaxis
	**CVS**	**C**ardio**V**ascular **S**ystem infections: endocarditis, endovascular prosthesis or device e.g pacemaker, vascular graft
**GI**	**Proph GI**	Surgery of the **G**astro-**I**ntestinal tract, liver or biliary tree, GI prophylaxis in neutropaenic patients or hepatic failure
	**GI**	GI infections (salmonellosis, *Campylobacter*, parasitic, *C.difficile*, etc.)
	**IA**	**I**ntra-**A**bdominal sepsis including hepatobiliary, intra-abdominal abscess *etc*.
**SSTBJ**	**Proph BJ**	Prophylaxis for SST, for plastic or orthopaedic surgery (**B**one or **J**oint)
	**SST**	**S**kin and **S**oft **T**issue: Cellulitis, wound including surgical site infection, deep soft tissue not involving bone e.g., infected pressure or diabetic ulcer, abscess
	**BJ**	**B**one/**J**oint Infections: Septic arthritis (including prosthetic joint), osteomyelitis
**UTI**	**Proph UTI**	Prophylaxis for urological surgery **(SP)** or recurrent **U**rinary **T**ract **I**nfection **(MP)**
	**Cys**	Lower UTI
	**Pye**	Upper UTI including catheter related urinary tract infection, pyelonephritis
**GUOB**	**Proph OBGY**	Prophylaxis for **OB**stetric or **GY**naecological surgery
	**OBGY**	**Ob**stetric/**Gy**naecological infections, **S**exual **T**ransmitted **D**iseases (**STD**) in women
	**GUM**	**G**enito-**U**rinary **M**ales + Prostatitis, epididymo-orchitis, STD in men
**No defined site (NDS)**	**BAC**	Bacteraemia with no clear anatomic site and no shock
	**SEPSIS**	Sepsis, sepsis syndrome or septic shock with no clear anatomic site
	**Malaria**	
	**HIV**	Human immunodeficiency virus
	**PUO**	**P**yrexia of **U**nknown **O**rigin - Fever syndrome with no identified source or site of infection
	**PUO-HO**	Fever syndrome in the non-neutropaenic **H**aematology–**O**ncolgy patient with no identified source of pathogen
	**FN**	**F**ever in the **N**eutropenic patient
	**LYMPH**	Infection of the **lymph**atics as the primary source of infection e.g.suppurative lymphadenitis
	**Other**	Antibiotic prescribed with documentation for which there is no above diagnosis group
	**MP-GEN**	Drug is used as **M**edical **P**rophylaxis in **gen**eral, without targeting a specific site, e.g. antifungal prophylaxis during immunosuppression
	**UNK**	Completely **Unk**nown Indication
	**PROK**	Antimicrobial (e.g. erythromycin) prescribed for **Prok**inetic use
**Neonatal**	**MP-MAT**	Drug is used as **M**edical **P**rophylaxis for **MAT**ERNAL risk factors e.g. maternal prolonged rupture of membranes
	**NEO-MP**	Drug is used as **M**edical **P**rophylaxis for **NE**WBORN risk factors e.g. VLBW (Very Low Birth Weight) and IUGR (Intrauterine Growth Restriction)

APPENDIX III - Type of Indication

<table>
<tr><td><u>CAI</u> Community acquired infection</td><td colspan="3">Symptoms started <48 hours from admission to hospital (or present on admission).</td></tr>
<tr><td rowspan="6"><u>HAI</u> Healthcare-Associated Infection
➢ Symptoms start 48 hours after admission to hospital</td><td colspan="3"><u>HAI1</u> Post-operative surgical site infection (within: 30 days of surgery OR; 1 year after implant surgery)</td></tr>
<tr><td colspan="3"><u>HAI2</u> Intervention related infections including CR-BSI, VAP and C-UTI</td></tr>
<tr><td colspan="3"><u>HAI3</u> C. difficile associated diarrhoea (CDAD) (>48 h post-admission or <30 days after discharge from previous admission episode.</td></tr>
<tr><td colspan="3"><u>HAI4</u> Other hospital acquired infection (includes HAP, etc)</td></tr>
<tr><td colspan="3"><u>HAI5</u> Infection present on admission from another hospital (patient with infection from another hospital)</td></tr>
<tr><td colspan="3"><u>HAI6</u> Infection present on admission from long-term care facility (LTCF) or Nursing Home*.</td></tr>
<tr><td><u>SP</u> Surgical prophylaxis</td><td><u>SP1</u> Single dose</td><td><u>SP2</u> one day</td><td><u>SP3</u> >1 day</td></tr>
<tr><td colspan="4">For surgical patients, administration of prophylactic antimicrobials should be checked in the previous 24 hours in order to encode the duration of prophylaxis as either one dose, one day (= multiple doses given within 24 hours) or >1 day.
See more explanation in protocol page 6 and 7 !</td></tr>
<tr><td><u>MP</u> Medical prophylaxis</td><td colspan="3">For example long term use to prevent UTI’s or use of antifungals in patients undergoing chemotherapy or penicillin in asplenic patients etc.</td></tr>
<tr><td><u>OTH</u> Other</td><td colspan="3">For example erythromycin as a motility agent (motilin agonist).</td></tr>
<tr><td><u>UNK</u></td><td colspan="3">Completely unknown indication</td></tr>
</table>

Select 1 possibility for each reported antimicrobial

CR-BSI= Catheter related-Blood Stream Infection
C-UTI= Catheter related-Urinary Tract Infection
HAP=Hospital Acquired Pneumonia
VAP=Ventilator Associated Pneumonia

* Long-term care facilities represent a heterogeneous group of healthcare facilities, with care ranging from social to medical care. These are places of collective living where care and accommodation is provided as a package by a public-agency, non-profit or private company (e.g. nursing homes, residential homes).
